# Maternal and neonatal outcomes of cancer during pregnancy: a multi-center observational study

**DOI:** 10.7150/jca.33746

**Published:** 2019-10-03

**Authors:** Zhang YP, Duan J, Zhu XW, Li J, Shi Y

**Affiliations:** 1 Department of Pediatrics, The Second Affiliated Hospital of the Third Military Medical University, Chongqing, 400037 China.; 2 Department of Pediatrics, the People's Hospital of Shapingba District, Chongqing, 400030, China.; 3 Department of Neonatology, Jiulongpo People's Hospital, Chongqing, 400024, China; 4 Department of Obstetrics and Gynecology, The First Affiliated Hospital of Chongqing Medical University, Chongqing, 400014, China.; 5 China International Science and Technology Cooperation base of Child development and Critical Disorders; Chongqing Key Laboratory of Pediatrics, Children's Hospital of Chongqing Medical University, Chongqing, 400014, P.R China.

**Keywords:** pregnancy, complicating cancer

## Abstract

Cancer during pregnancy has increased because of the increased maternal age at the time of the first pregnancy and/or second child policy in China. The main purpose of the study is to report the existing data concerning the maternal and children's outcomes in pregnant women complicating cancer. In this multi-center, prospective cohort study, we compared women diagnosed with cancer during pregnancy and their children with matched women without cancer diagnoses. The primary outcomes were maternal and children's mortalities, as well as the Ages and Stages Questionnaires-3(ASQ) of children. A total of 80,524 pregnant women were screened. Of whom 83(0.1%) were diagnosed with cancer during pregnancy. Death occurs in 42.2% (35/83) women during follow-up. During pregnancy, 24 women terminated pregnancy before 28 weeks and among this 8(33.3%) died. Ten women received chemotherapy and 49 did not receive chemotherapy. Compared with the matched controls, there were less incidences of premature rupture of membrane (5.1% vs 35.6%, *P*=0.000) and more caesarean rates (84.7% vs 52.5%, *P*=0.001) and with higher pregnancy order (2.7±1.7 vs 2.0±1.0, *P*=0.007) in pregnant women with cancer. These women also had increased mortality compared with control group (45.8% vs 1.7%, *P*=0.000). Women who received chemotherapy had a statistically significant lower mortality rate when compared to the non-chemotherapy group (1:9 vs 26:23, *P*=0.016). However, there were no differences found in mortality of children and ASQ assessment between two groups. Chemotherapy may result in reduced mortality of women diagnosed with cancer during pregnancy, without affecting the mortality of children and ASQ-associated development.

## Introduction

Cancer during pregnancy is uncommon and occurs in about 1 per 1000 pregnancies 1. With more women delaying childbearing to later maternal ages and/or responding to two-child policy in 2016 in China, the rates of cancer during pregnancy will likely continue to increase^2^. Once cancer during pregnancy is diagnosed, decision whether to terminate the pregnancy before initiating treatment or to continue pregnancy becomes important. Terminating pregnancy is primarily for maternal health reasons. If pregnancy is continued, there is the potential for chemotherapeutic agents causing fetal damage during the period of rapid growth.

However, pregnant women diagnosed with breast cancer who have received treatment compared to non-pregnant women lead to a similar survival^3^. In contrast, terminating pregnancy did not produce a survival benefit in pregnant women diagnosed with breast cancer, one of the most common malignancies^4^. Many chemotherapeutic drugs which are toxic can pass through the placenta, and influence cell division of fetus. This may inhibit fetal and subsequently neonatal development. However, the majority of children who were exposed to chemotherapy in utero did not demonstrate significant complications^3^. A multi-center study also showed that delayed termination of pregnancy or cancer treatment did not impair the cognitive, cardiac, or general development of children in early childhood^5^.

Limited data are available regarding cancer diagnosis during pregnancy, treatment options and outcomes in China. Therefore, the main aim of the present study is to describe the available data on women with cancer diagnosed during pregnancy, maternal outcomes and their children's short-term and long-term outcomes.

## Materials and Methods

### Study Design and Participants

This was a prospective, multi-center cohort study conducted in Daping Hospital, Xinqiao Hospital and Xinan Hospital of Third Military Medical University from Jan, 2012 to Jan, 2017. The study was approved by the Ethics Committee of Daping Hospital and research institute of surgery and registered at http://www.chictr.org.cn. (ID: CHiCTR-ERC-17012146). It was from a prospective protocol, and informed parental written consents were obtained prior to study. The trial was performed in accordance with the approved guidelines and regulations of the participating institutions.

Inclusion criteria were women diagnosed with any cancers during pregnancy with or without therapies during pregnancy, and the cancer was diagnosed according to histological examination and TNM system used for tumors staging. Exclusion criteria were: (1) parents' decision not to participate. (2) Diagnosed with cancer before and after pregnancy.

Control children were matched in a 1:1 ratio and must meet four criteria simultaneously: (1) Born to pregnant women without cancer in the same hospital; (2) Mothers adjacent to the pregnant women with cancer among the mothers satisfied matched conditions; (3) Mothers with similar infants that are gestational age and less than three days and (4) Mothers with the similar other pregnancy complications at birth.

### Data Collection

The clinical data of all enrolled pregnant women and their neonates were collected, including obstetrical care, neonatal, and oncologic data in standardized case report forms.

We also obtained the living conditions on women and their children. If the children were alive, the Ages and Stages Questionnaires-3(ASQ) scores of children were recorded either by face-to-face or telephone testing.

### Evaluation of primary and secondary outcomes

The primary outcomes of this study were to determine the mortality of women with cancer and their children, and the performances in ASQ. The secondary outcomes were to determine the rates of premature rupture of membranes, caesarean delivery, birth asphyxia, and respiratory complications.

### The Ages and Stages Questionnaires (ASQ)

The children' development was assessed by the Chinese version of the Ages and Stages Questionnaire(ASQ) 6,7. According to the Chinese manual, the ASQ is a parent-done, developmental screening measure, in which parents answer 30 questions covering 5 domains of development, including communication, gross motor, fine motor, problem-solving, and adaptive skills. And 20 age-associated questionnaires intended for use from the age of 1 month to 66 months. Parents were instructed to assess if their child could finished task: 10 points for “yes”, 5 points for “sometimes” or 0 points for “not yet”. Based on the scores, the children were divided into 3 grades: (1) Abnormal development means that the score of any domain screened is less than mean-2 standard deviation. (2) Borderline development means that the score is between mean-2 standard deviation and mean-standard deviation. (3) Normal development means that the score is more than mean-standard deviation^6^-^8^.

### Statistical analysis

Continuous variables, expressed as mean ± standard deviation, were compared using paired samples t test or independent samples t test. Pair-wise comparisons were evaluated by the Student-Newman-Keuls procedure. Categorical paired variables were compared using the McNemar's test, the others were compared using the χ2 test or the Fisher's test. All analyses were carried out using computer software (SPSS 16.0 for windows). A *p*-values less than 0.05 was regarded as statistically significant.

## Results

### Baseline characteristics

**Figure 1:**
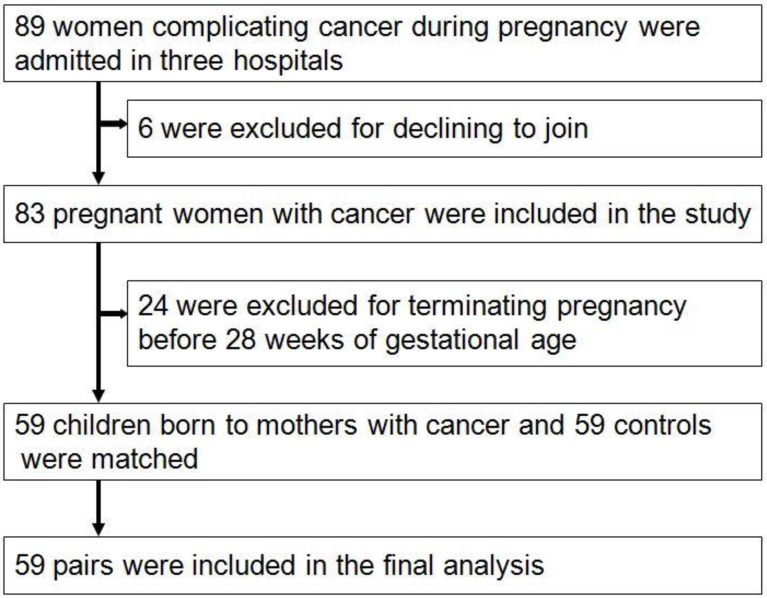
The flow diagram of study design and recruitment

From Jan, 2012 to Dec, 2016, a total of 80,524 pregnant women were screened (32,263 came from Xinan Hospital, 23,692 came from Xinqiao Hospital and 24,569 came from Daping Hospital, respectively). Among these 83 were diagnosed with cancer during pregnancy, and the rate of cancer during pregnancy was 0.1% (83/80524). Thirty-five women died during the follow-up, and the rates of mortality were 42.2%(35/83). The main five cancers were leukemia, thyroid carcinoma, cervical carcinoma, ovarian cancer and breast cancer. During pregnancy, 10 women received chemotherapy, no women received radiotherapy, and 49 did not receive chemotherapy until delivery. Twenty-four pregnant women, with a mean age of 29.9±6.5 years, terminated the pregnancy before 28 weeks and received the cancer-associated treatment. Among these women, 8(33.3%) died with mean follow-up time of 37.0±20.0 months. Of them, 19 came from country area, and other 5 came from urban area. Finally, 59 pregnant women with cancer were included, with mean follow-up time of 29.7±17.8 months, and 59 neonates were matched as controls and finished the analysis (Fig. 1). The main clinical characteristics and outcomes of women diagnosed with cancer during pregnancy and the matched controls are shown in table 1.

### Primary outcome

The ASQ was given to 118 parents or women complicating cancer during pregnancy and the matched controls. 5 children of women complicating cancer during pregnancy and 3 controls died, respectively. Finally, we received 110 feedback(54 in cancer group and 56 in control group). Of 54 children of women complicating cancer during pregnancy, 48 were shown normal development and 6 borderline developments, respectively. Of 56 controls, 49 were shown normal development and 7 borderline developments, respectively. No children were shown abnormal development.

Pregnant women in cancer group had an increased mortality rate as compared to the control group (45.8% vs 1.7%, *P*=0.000). Otherwise, compared with continuing pregnancy(n=59), terminating pregnancy(n=24) did not result in reduced maternal mortality (45.8% vs 33.3%, P=0.336 ).

Women who received chemotherapy had a statistically significant lower mortality rate when compared to the non-treatment group (1:9 vs 26:23, *P*=0.016) (Table 2). However, The mortality of children(1:9 vs 4:45, *P*=1.000) and ASQ assessment(8:1 vs 39:6, *P*=1.000) were not difference between two groups (Table 2).

Compared with the neonates from the matched pregnant women, there were no differences in children death (8.5% vs 5.1%, *P*=0.717) and ASQ score (88.9% vs 87.5%, *P*=1.000) (Table 1).

### Secondary outcomes

Of 59 pregnant women with cancer, the rates of preterm premature rupture of the membrane was 3.61%, pregnancy induced diabetes was 4.82%, and intrahepatic cholestasis of pregnancy was 1.20% respectively. Cesarean section rates were high with 21 (35.6%) infants born via urgent cesarean delivery, 29 (49.1%) born via elective cesarean delivery, and the 9 (15.3%) born via vaginal delivery.

Compared with the matched controls, there were less preterm rupture of membrane(5.1% vs 35.6%, *P*=0.000) and more caesarean section(50:9 vs 31:28, *P*=0.001) in pregnant women with cancer. Pregnant women in cancer group had higher maternal age (30.6±5.3 vs 28.2±3.2 yrs, *P*=0.005) and higher pregnant numbers (2.7±1.7 vs 2.0±1.0, *P*=0.007). Besides, there were less first pregnancy in the pregnant women with cancer (64.4% vs 72.9%, *P*=0.008). However, the 95% confidence interval covered 1 (0.308-1.474). There were no differences in the clinical characteristics of the neonates at birth between two groups. Compared with the neonates from the matched pregnant women, there were no differences in short-term and long-term outcomes, including asphyxia (11.9% vs 6.8%, *P*=0.528) and respiratory failure (23.7% vs 27.1%, *P*=0.833) (Table 1).

### Subgroup Analyses

Chemotherapy was administered to 10 women during the second and third trimesters. No women received chemotherapy during the first trimester. There were no chemotherapy-associated neonatal malformations.

## Discussion

In this multi-center, prospective cohort study, we aimed to analyze the existing data regarding the maternal and children's outcomes, of mother with cancer diagnosis during pregnancy. Our observations suggest that chemotherapy may be feasible and safe during the second and third trimester pregnancy and improve the prognosis of women, and no adverse effects related to the chemotherapy were seen in both women and their children.

Previous several studies have compared the effects of cancer during pregnancy on the growth and development of children, and the results are inconsistent. Cardonick E et al.^3^ showed that the majority of children who were exposed to chemotherapy in utero did not demonstrate significant complications, and Murthy RK et al. 9 reported that, during the second and third trimesters, pregnant women with breast cancer can be treated with 5-fluorouracil, doxorubicin, and cyclophosphamide safely without concerns for serious complications or short-term health concerns for their offspring who were exposed to chemotherapy in utero. With regards to long-term outcomes, Amant F et al. reported that fetal exposure to chemotherapy was not associated with increased central nervous system, cardiac or auditory morbidity, or with impairments to general health and growth compared with the general population^10^. Similar result was reported by Abdel-Hady el-S, et al. 11. Recently, the multi-centered study that compared children of mothers with cancer during the pregnancy with matched children of women without a cancer also showed that prenatal exposure to maternal cancer with or without treatment did not impair the cognitive, cardiac, or general development of children in early childhood^5^. Our data is consistent with the reports of Gwyn K. 12 and Hahn KM et al. 13. Where cancer with and without chemotherapy administered during the second and third trimester does not seem to affect both short and long term development of children^14^. In contrast, previous studies demonstrated chemotherapy during embryogenesis increased risks of spontaneous abortions and major birth defects^15^ and stillbirth, fetal growth restriction, premature birth, and maternal and fetal myelosuppression^16^.

Chemotherapy during pregnancy can result in significant side effects to the fetus, the children, and their mothers. For the fetus and the children, these harmful effects include malformations, organ toxicity, death and delayed development if they survive; For the mothers, the death due to delayed treatment can occur at a rate of 1%-3%^17^. Chemotherapy administrated early in pregnancy increased fetal malformation to 12.7%-17%, as well as low birth weight by 40%^1^. In the present study, chemotherapy was administratered to 10 women during the second and third trimesters. As a result, there were no chemotherapy-associated neonatal malformations. In addition, there were no difference in body weight, death and ASQ assessment in the treatment group in consistent with previous reports of. Cardonick et al. 3 demonstrated that pregnant women diagnosed with breast cancer receiving chemotherapy have similar survival rates when matched for stage at the time of diagnosis. Furthermore, Yang WT, et al. 18 has shown that compared with non-pregnant women, there was no significant difference at each stage of 5-year survival in pregnant women. These observations are consistent with our findings where chemotherapy seems to improve survival in pregnancy complicated by cancer diagnosis.

In our population compared with continuing pregnancy, terminating pregnancy did not result in reduced maternal mortality (8/16 vs 27/32, P=0.336), and which was consistent to several previous reports of Zemlickis D, et al. 19 and Framarino-Dei-Malatesta M, et al.^20^ Based on these observations immediate termination may not be routinely needed as the maternal and fetal outcomes did not appear to be compromised if chemotherapy is delayed to second trimester and beyond^21^. In contrast, Deemarsky LJ, et al. 22 reported a worse prognosis for 14 women who terminated the pregnancy, compared with 8 who continued the pregnancy. Another study also showed that, compared with not pregnant women, there was a higher mortality in pregnant women^23^.

One of the possible causes to explain the inconsistence could be the different types and stages of cancer among different studies. As is known, there are statistically significant differences in survival among the different stages of cancer. The stage distribution of the controls was statistically significantly different from that of the pregnant women (0.025 < *P* < 0.05) in the results of Zemlickis D, et al. 19 However, Framarino-Dei-Malatesta M, et al. 20 did not demonstrate differences in any stages and grades of cancer. Further analysis^19^ found that, compared with the controls, there were statistically lower number of women diagnosed with stage I disease *(P*=0.015) and higher proportion of women diagnosed with stage IV*(P*=0.013) in the pregnant group.

Another study in China demonstrated increased preterm rupture of membrane and caesarean section in women complicating cancer during pregnancy^24^. It was similar to our observations. We conferred that the women complicating cancer during pregnancy was nervous and anxious, and they were inclined to terminate pregnancy earlier and initiate treatment in the premise of avoiding toxicities of chemotherapy to fetus.

In the present study, the five main cancers were leukemia, thyroid carcinoma, cervical carcinoma, oophoroma and breast cancer. This is different from the report of Pavlidis NA.^1^, where the most common malignancies associated with pregnancy included cervical cancer, breast cancer, melanoma, lymphomas, and leukemia.

In summary, our observations suggest that chemotherapy administrated in second and third trimesters may maximize the benefits of both the mothers and their children. Given the side effects of chemotherapy drugs and heterogeneities of cancer types, more similar study and further follow-up of children are needed. Recently, we have organized a multi-centers trial included 83 hospitals, the maternal age and single tumor and their TNM stage were matched in the study and the aim was to describe the available data on women with and without cancers diagnosed during pregnancy. Maternal outcomes and their children's short-term and long-term outcomes, and the results may give us more reasonable justifications.

## Figures and Tables

**Figure 2:**
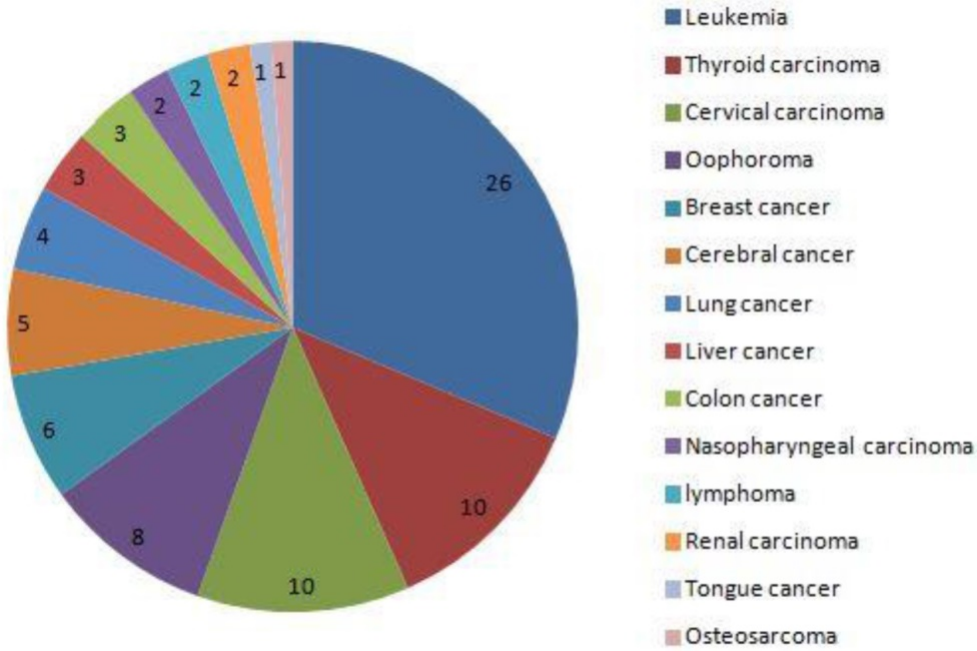
The distribution of cancer in 83 pregnant women. the 5 main cancers was leukemia, thyroid carcinoma, cervical carcinoma, oophoroma and breast cancer.

**Figure 3:**
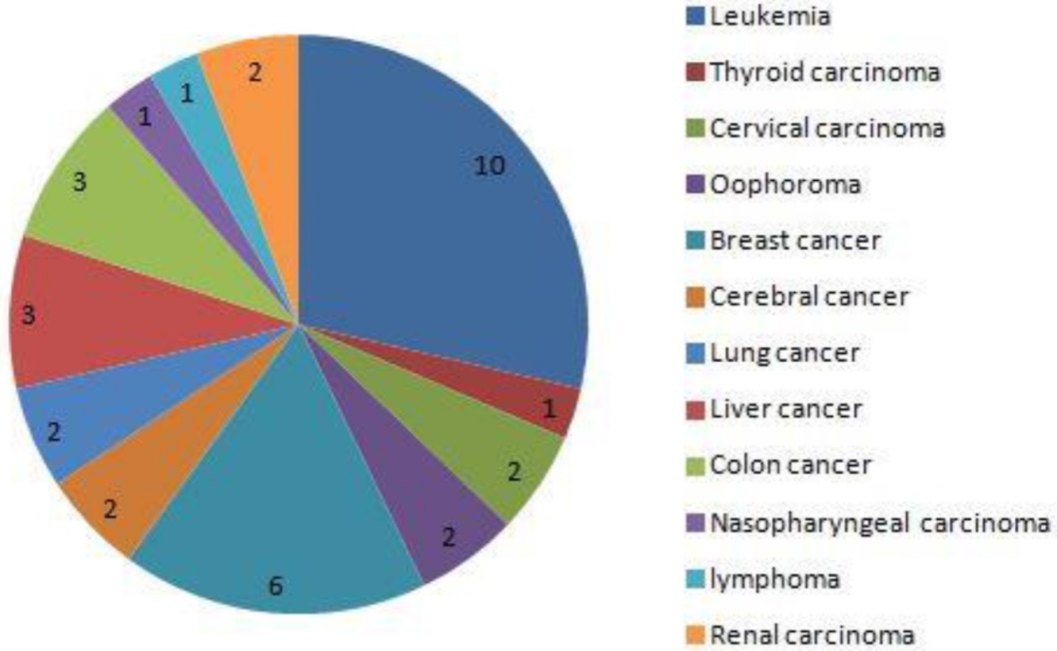
The distribution of cancer in 35 dead women. the 5 main cancers was leukemia, thyroid carcinoma, cervical carcinoma, oophoroma and breast cancer.

**Figure 4:**
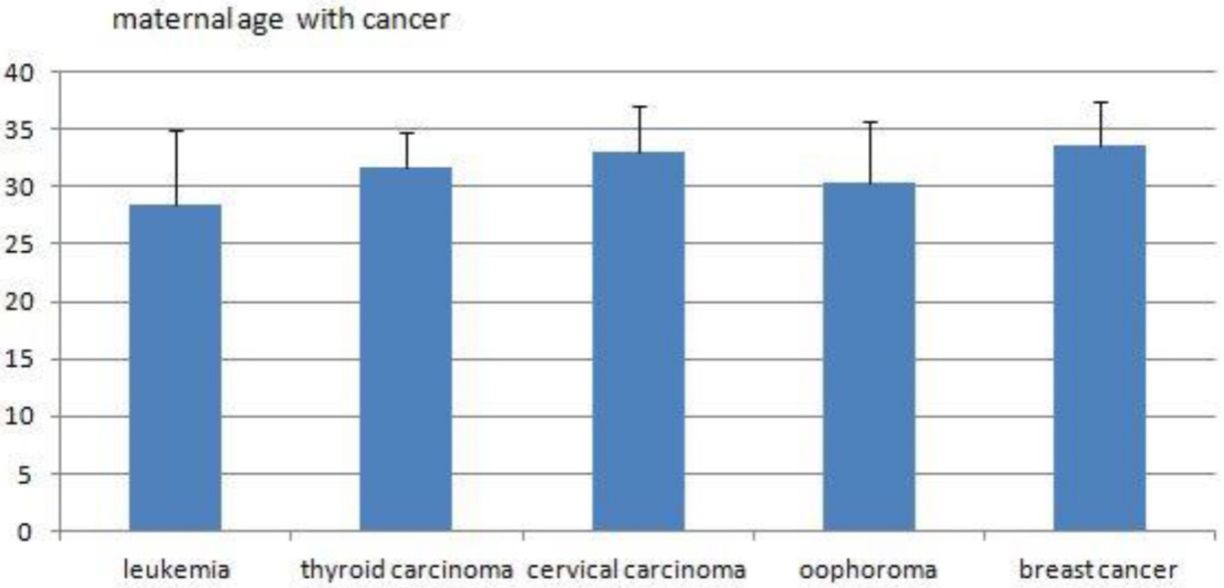
The average maternal ages of the five main cancers.

**Figure 5:**
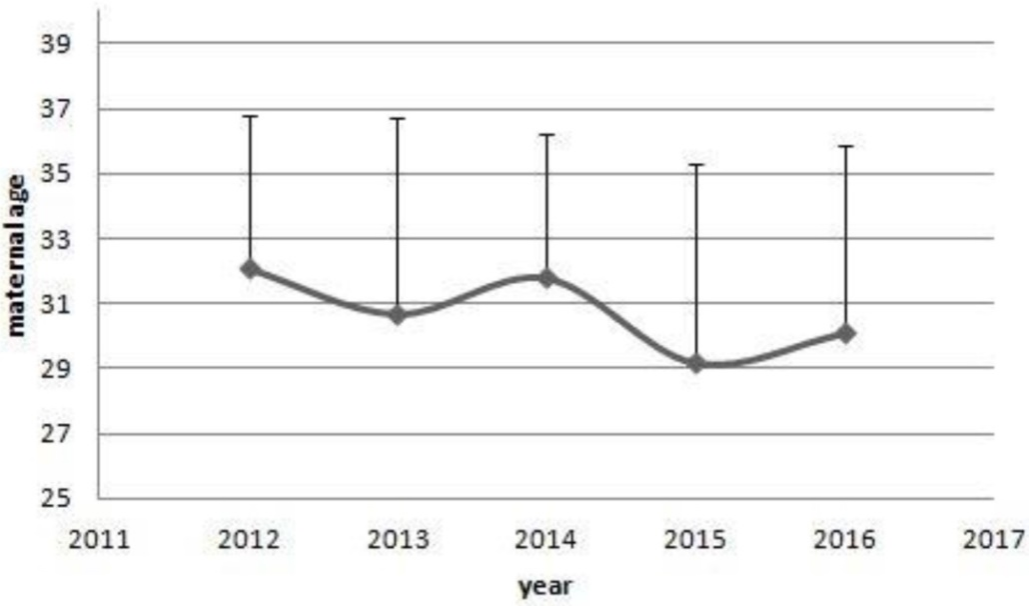
The trends of maternal age of women with cancer. From 2012 to 2016, there were a decreased trends in the maternal age of women with cancer diagnosed. However, no significant differences were shown among years. (32.1±4.7 vs 30.7±6.0 vs 31.8±4.4 vs 29.2±6.1 vs 30.1±5.8, *P*=0.644)

**Figure 6:**
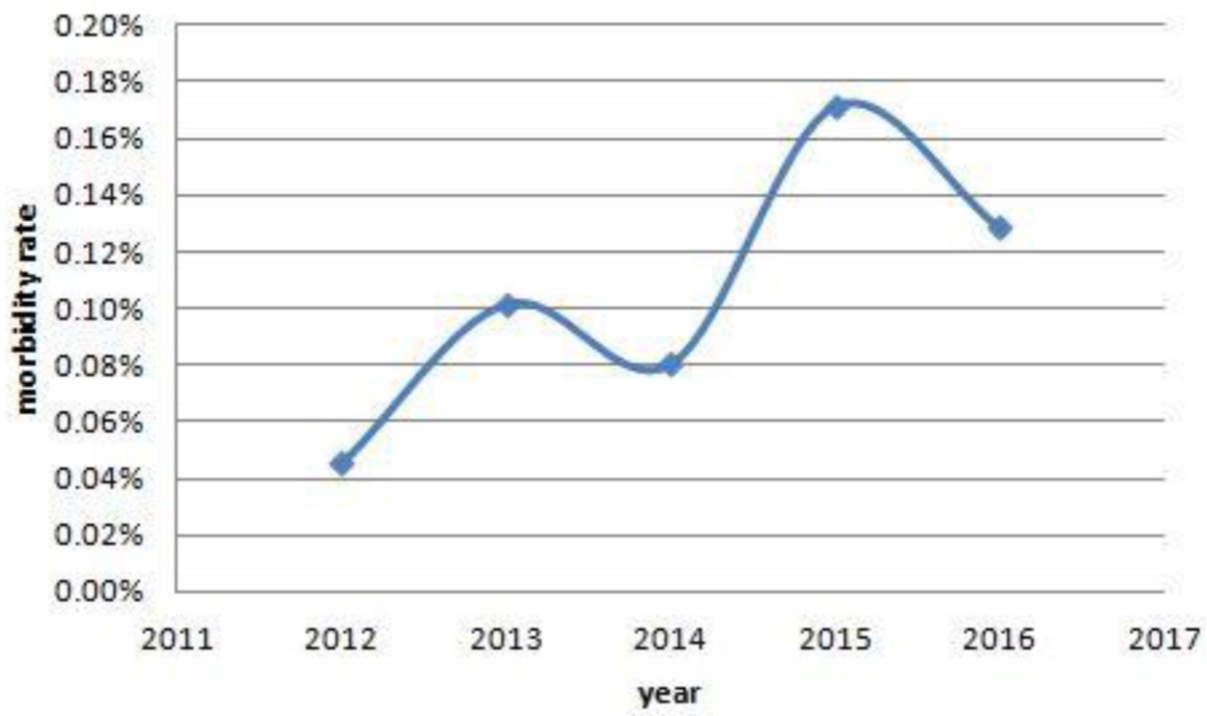
The trends of morbidity rate of women with cancer. From 2012 to 2016, there were a increased trends in the morbidity rate of women with cancer, and they were 7/15404, 16/15712, 12/14890, 25/14550, 23/17900, respectively. Also, significant differences were found among the past five years. (0.05% vs 0.10% vs 0.08% vs 0.17% vs 0.13%, *P*=0.010).

**Table 1:** Clinical characteristics and outcomes of women diagnosed with cancer during pregnancy in 59 continuing pregnancy and the matched women without cancer.

	Cancer group(n=59)	Control group(n=59)	95%CI	P-value
Maternal age (years)	30.6±5.3	28.2±3.2	0.724-3.920	0.005
Pregnant numbers	2.7±1.7	2.0±1.0	0.190-1.132	0.007
The first pregnancy (yes: no)	38:21	43:16	0.308-1.474	0.008
PROM (yes: no)	3:56	21:38	0.027-0.348	0.000
Caesarean (yes: no)	50:9	31:28	2.093-12.031	0.001
Antenatal glucocorticoids (yes: no)	21:38	26:33	0.335-1.471	0.169
Maternal death (yes: no)	27:32	1:58	6.350-377.136	0.000
Gestational age (weeks)	260.6±22.1	260.0±22.3	(-7.535)-8.653	0.891
Gender (m: f)	32:27	39:20	0.289-1.278	0.175
Birth weight (g)	2831.9±762.6	2766.8±791.1	(-201.548)-331.819	0.627
Apgar 1 min	9.1±2.3	9.3±1.3	(-0.978)-0.469	0.485
Apgar 5 min	9.1±2.6	9.6±1.3	(-1.275)-0.292	0.214
Apgar 10 min	9.2±2.4	9.5±1.6	(-1.049)-0.507	0.488
Urban: country	23:36	43:16	0.109-0.517	0.500
Asphyxia	7:52	4:55	0.512-6.695	0.528
Respiratory failure (yes: no)	14:45	16:43	0.365-1.918	0.833
Children death (yes no)	5:54	3:56	0.394-7.588	0.717
ASQ score	48:6	49:7	0.358-3.649	1.000

**Table 2:** The primary and secondary outcomes of women and their children in 59 continuing pregnancy.

	Treatment group(n=10)	Non-treatment group(n=49)	95%CI	P-value
Maternal age (years)	29.2±2.7	30.8±5.6	(-4.02)-0.74	0.170
Gestational age (days)	250.9±21.6	261.9±21.9	(-26.21)-4.13	0.151
Gender (m: f)	7:3	25:24	0.518-9.683	0.319
Birth weight (g)	2493.0±682.2	2901.1±766.0	(-931.59)-115.35	0.124
Pregnant numbers	3.1±1.5	2.7±1.7	(-0.739)-1.633	0.454
The first pregnancy (yes: no)	6:4	32:17	0.197-3.216	0.733
Premature (yes: no)	3:7	16:33	0.202-3.877	1.000
PROM (yes: no)	0:10	3:46	0.992-1.144	1.000
Antenatal glucocorticoids (yes: no)	6:4	15:34	0.836-13.835	0.144
Caesarean (yes: no)	9:1	41:8	0.194-15.857	1.000
Apgar 1 min	9.1±2.2	9.0±2.3	(-1.56)-1.59	0.982
Apgar 5 min	8.9±3.1	9.1±2.5	(-2.02)-1.57	0.805
Apgar 10 min	8.9±3.1	9.3±2.2	(-2.05)-1.28	0.644
Urban: country	5:5	18:31	0.438-6.770	0.490
Asphyxia (yes: no)	1:9	6:43	0.085-7.447	1.000
Respiratory failure (yes: no)	2:8	12:37	0.144-4.139	1.000
Maternal death (yes: no)	1:9	26:23	0.012-0.836	0.016
Children death (yes no)	1:9	4:45	0.125-12.533	1.000
ASQ score	8:1	39:6	0.130-11.672	1.000
